# The double-stranded RNA-binding protein, Staufen1, is an IRES-transacting factor regulating HIV-1 cap-independent translation initiation

**DOI:** 10.1093/nar/gkab1188

**Published:** 2021-12-06

**Authors:** Hade Ramos, Anne Monette, Meijuan Niu, Aldo Barrera, Brenda López-Ulloa, Yazmín Fuentes, Paola Guizar, Karla Pino, Luc DesGroseillers, Andrew J Mouland, Marcelo López-Lastra

**Affiliations:** Laboratorio de Virología Molecular, Instituto Milenio de Inmunología e Inmunoterapia, Departamento de Enfermedades Infecciosas e Inmunología Pediátrica, Escuela de Medicina, Pontificia Universidad Católica de Chile, Marcoleta 391, Santiago, Chile; HIV-1 RNA Trafficking Laboratory, Lady Davis Institute at the Jewish General Hospital, Montréal, Québec H3T 1E2, Canada; HIV-1 RNA Trafficking Laboratory, Lady Davis Institute at the Jewish General Hospital, Montréal, Québec H3T 1E2, Canada; Laboratorio de Virología Molecular, Instituto Milenio de Inmunología e Inmunoterapia, Departamento de Enfermedades Infecciosas e Inmunología Pediátrica, Escuela de Medicina, Pontificia Universidad Católica de Chile, Marcoleta 391, Santiago, Chile; Laboratorio de Virología Molecular, Instituto Milenio de Inmunología e Inmunoterapia, Departamento de Enfermedades Infecciosas e Inmunología Pediátrica, Escuela de Medicina, Pontificia Universidad Católica de Chile, Marcoleta 391, Santiago, Chile; Laboratorio de Virología Molecular, Instituto Milenio de Inmunología e Inmunoterapia, Departamento de Enfermedades Infecciosas e Inmunología Pediátrica, Escuela de Medicina, Pontificia Universidad Católica de Chile, Marcoleta 391, Santiago, Chile; HIV-1 RNA Trafficking Laboratory, Lady Davis Institute at the Jewish General Hospital, Montréal, Québec H3T 1E2, Canada; Department of Medicine, McGill University, Montréal, Québec H4A 3J1, Canada; Laboratorio de Virología Molecular, Instituto Milenio de Inmunología e Inmunoterapia, Departamento de Enfermedades Infecciosas e Inmunología Pediátrica, Escuela de Medicina, Pontificia Universidad Católica de Chile, Marcoleta 391, Santiago, Chile; Department of Biochemistry and Molecular Medicine, University of Montreal, P.O. Box 6128, Station Centre Ville, Montreal, Québec H3C 3J7, Canada; HIV-1 RNA Trafficking Laboratory, Lady Davis Institute at the Jewish General Hospital, Montréal, Québec H3T 1E2, Canada; Department of Medicine, McGill University, Montréal, Québec H4A 3J1, Canada; Laboratorio de Virología Molecular, Instituto Milenio de Inmunología e Inmunoterapia, Departamento de Enfermedades Infecciosas e Inmunología Pediátrica, Escuela de Medicina, Pontificia Universidad Católica de Chile, Marcoleta 391, Santiago, Chile

## Abstract

Translation initiation of the viral genomic mRNA (vRNA) of human immunodeficiency virus-type 1 (HIV-1) can be mediated by a cap- or an internal ribosome entry site (IRES)-dependent mechanism. A previous report shows that Staufen1, a cellular double-stranded (ds) RNA-binding protein (RBP), binds to the 5’untranslated region (5′UTR) of the HIV-1 vRNA and promotes its cap-dependent translation. In this study, we now evaluate the role of Staufen1 as an HIV-1 IRES-transacting factor (ITAF). We first confirm that Staufen1 associates with both the HIV-1 vRNA and the Gag protein during HIV-1 replication. We found that in HIV-1-expressing cells, siRNA-mediated depletion of Staufen1 reduces HIV-1 vRNA translation. Using dual-luciferase bicistronic mRNAs, we show that the siRNA-mediated depletion and cDNA-mediated overexpression of Staufen1 acutely regulates HIV-1 IRES activity. Furthermore, we show that Staufen1-vRNA interaction is required for the enhancement of HIV-1 IRES activity. Interestingly, we find that only Staufen1 harboring an intact dsRNA-binding domain 3 (dsRBD3) rescues HIV-1 IRES activity in Staufen1 CRISPR-Cas9 gene edited cells. Finally, we show that the expression of Staufen1-dsRBD3 alone enhances HIV-1 IRES activity. This study provides evidence of a novel role for Staufen1 as an ITAF promoting HIV-1 vRNA IRES activity.

## INTRODUCTION

The eukaryotic messenger RNAs (mRNAs) translation process comprises the initiation, elongation, termination and ribosome recycling steps (1–3). Though each of these stages can be tightly regulated to impact the rate of protein synthesis, mRNA translation is mainly controlled at the initiation step. Most eukaryotic mRNAs rely on the recognition of their 5′end m^7^GpppN (7-methylguanosine; cap) for translation initiation (1,2). In cap-dependent translation initiation, the eukaryotic initiation factor (eIF) 4F heterotrimeric complex comprising eIF4E, eIF4A and eIF4G, recognizes the 5′cap- driving 40S ribosomal subunit recruitment to the mRNA (3). The cap-binding protein eIF4E recognizes the cap-structure, eIF4A, an ATP-dependent RNA helicase, unwinds RNA structures present within the 5′untranslated region (UTR) of the mRNA, while eIF4G acts as a scaffold bridging eIF4E, eIF4A and the 40S ribosomal subunit (via eIF3) (3). Upon recruitment, the 40S ribosomal subunit scans the mRNA in a 5′-3′ direction until the initiation codon is encountered, leading to the joining of the 60S ribosomal subunit to assemble a translationally competent 80S ribosome (1,2). Under normal physiological conditions, translation initiation of cellular mRNAs is mostly cap-dependent (1,2,4,5). Approximately 10% of all cellular mRNAs also harbor an RNA element termed the internal ribosome entry site (IRES), enabling cap-independent internal recruitment of the 40S ribosomal subunit to the mRNA (4–7). In cells, IRES-mediated initiation predominates under physiological conditions that suppress cap-dependent translation (4,5). Retroviruses, including the human immunodeficiency virus-type 1 (HIV-1), the major causative agent of the acquired immune deficiency syndrome (AIDS) pandemic, have adopted multiple strategies to guarantee viral protein synthesis (8,9). For example, translation initiation of the 9 kb HIV-1 genomic RNA (vRNA) occurs via cap-dependent (10–15) or IRES-dependent mechanisms (12,16–19). The importance of these alternative initiation mechanisms is underscored by earlier findings indicating that the translation initiation of the HIV-1 vRNA becomes IRES-dependent when cellular cap-dependent translation initiation is compromised (12,20).

The 5′UTR of the HIV-1 vRNA comprises several well-defined stem-loops (SL), including the TAR and 5′poly(A) loops, the DIS, SD and packaging sequences, psi (21). The 5′UTR of the HIV-1 vRNA also enables IRES-mediated translation initiation (12,16,18–20,22–24). To date, the precise molecular mechanisms driving and regulating HIV-1 IRES activity remain poorly understood (8,9). HIV-1 IRES activity does, however, partially rely on the ribosomal protein eS25(24), initiation factors eIF5A and eIF4A (23,25,26), as well as several IRES-transacting factors (ITAFs) (8,23,27), including the heterogeneous nuclear ribonucleoprotein A1 (hnRNP A1), the human Rev-interacting protein (hRIP), DDX3 and the Hu antigen R (HuR) (26–29). Using a pull-down strategy followed by mass spectrometry, several well-characterized cellular RBPs, including Staufen1 and some hnRNPs, were shown to bind to the 5′UTR of the HIV-1 vRNA (30). For the majority of these, however, their specific roles in the HIV-1 replication cycle and vRNA translation remain largely unknown (9,27,30). To further understand the molecular basis of HIV-1 IRES function, from the identified proteins (30), we were interested in Staufen1, a ubiquitously expressed double-stranded (ds) RBP, as it was already recognized as a host protein involved in multiple steps of HIV-1 replication (31–36). Nonetheless, no functional association between Staufen1 and HIV-1 IRES activity had been established.

Two Staufen1 isoforms exist, Staufen1^55^ (Stau1^55^) and Staufen1^63^ (Stau1^63^), generated through alternative splicing (37–39). In cells, Staufen1 isoforms are components of RNP complexes involved in post-transcriptional mechanisms that control cellular gene expression, including mRNA trafficking, localization, translation, and decay (40–46). Staufen1 associates with the rough endoplasmic reticulum (RER) and actively translating ribosomes (37,38,45,47). Staufen1 also regulates Enterovirus 71 (EV-A71) and hepatitis C virus (HCV) IRES activity (48,49). Interestingly, translation initiation of the *staufen1* mRNA can either follow a canonical cap-dependent mechanism or via an IRES (50). As a consequence, the expression of Staufen1 is maintained under physiological conditions that induces a molecular switch from cap- to IRES-dependent translation initiation (50,51). Staufen1-RNP complexes in the context of HIV-1 gene expression are involved in post-transcriptional mechanisms, including vRNA trafficking, transport, cap-dependent translation initiation, and encapsidation (31,33–36,52). In HIV-1 expressing cells, Stau1^55^ overexpression enhances vRNA translation, while the depletion of both Stau1^55^ and Stau1^63^, but not Stau1^63^ alone, leads to a significant decrease in intracellular Gag protein with no apparent impact on vRNA abundance (31,53). This observation suggests that Stau1^55^ is involved in the modulation of HIV-1 protein synthesis, which is in agreement with the ability of Stau1^55^ to bind and stimulate translation of RNAs containing the HIV-1 vRNA TAR-SL (33,54). Consistent with this possibility, Staufen1 expression levels increase during prometaphase (55), the cell cycle stage during which the HIV-1 IRES is fully active (16,27). Therefore, we predicted that the role of Stau1^55^ in vRNA translation was not restricted to cap-dependent translation initiation, as previously reported (33). Using dual-luciferase bicistronic mRNAs, we show that the siRNA-mediated depletion of Staufen1 and cDNA overexpression of Stau1^55^ acutely regulates HIV-1 IRES activity. Furthermore, we find that only Stau1^55^ harboring an intact dsRNA-binding domain 3 (dsRBD3) rescues HIV-1 IRES activity in cells in which Staufen1 is knocked out (SKO) by CRISPR-Cas9. Finally, we establish that the expression of Stau1^55^-dsRBD3 alone enhances HIV-1 IRES activity. This study provides evidence that Stau1^55^ contributes to non-canonical translation initiation of the HIV-1 vRNA, and acts as an ITAF for the HIV-1 IRES.

## MATERIALS AND METHODS

### Plasmids

The proviral HIV-1 clone pNL-4.3 (56) was obtained through the NIH AIDS Reagent Program, Division of AIDS, NIAID, NIH. The HIV-1 pNL-4.3-RLuc provirus was kindly provided by Dr R. Soto-Rifo (Programa de Virología, ICBM, Universidad de Chile) (57). The dual-luciferase (dl) plasmids dl HIV-1 IRES, harboring the 5′UTR of the HIV-1 vRNA (1-336), dl HIV-1 IRES 104–336, dl HIV-1 IRES 1–104 and ΔSV40 dl HIV-1 IRES were described in (16,19). The pcDNA3-RSV-Staufen^55^-HA, pcDNA3-RSV-Staufen^55^-GFP-topaz, pcDNA3-RSV-Staufen^F135A^-HA pcDNA3-RSV-Staufen^F135A^-GFP- topaz, pCMV-Staufen 1-YFP, pCMV-Staufen^F135A^-YFP, pcDNA3-RSV-Stau1^55^-dsRBD3, pcDNA3-RSV-Stau1^55^-4K-GFP-topaz have been previously described (38,47,58,59). The TAR stem loop deletion mutants were generated using the Thermo Fisher Scientific Phusion Site-Directed Mutagenesis Kit (#F-541, Thermo Fisher Scientific Inc. Life Technologies Inc., Carlsbad, CA, USA) using primers designed according to the manufacturer's protocol. The dl HIV-1 IRES TAR-mut1 (Δ nts 36–38) was generated with the primers forward 5′-GAGCTCTCTGGCTAACTAGGGAAC-3′ and reverse 5′-GGCTCAGATCTGGTCTAACCAGAGAGA-3′; TAR-mut2 (Δnts 32–34) was generated with the primers forward 5′-CTGGGAGCTCTCTGGCTAACTAGGG-3′ and reverse 5′-CAGATCTGGTCTAACCAGAGAGAC-3′; and TAR-mut3 (Δ nts 27–45) was generated with the primers forward 5′-CTGGCTAACTAGGGAACCCACTG-3′ and reverse 5′-CTGGTCTAACCAGAGAGACCG-3′. The polymerase chain reaction (PCR) assays were performed in a Veriti TM 96-well Thermal Cycler (#4375768, Thermo Fisher Scientific Inc.). The sequence of all constructs used in this study was verified (Psomagen Inc., Rockville, MD, USA).

### Cell culture and drug treatments

HEK 293T (ATCC, CRL-11268) and HeLa cells (ATCC) were grown in Dulbecco's modified Eagle's medium (DMEM; #SH30022, HyClone, GE Healthcare Life Sciences, Logan, UT, USA) containing 10% fetal bovine serum (#SH30910, Hyclone, GE Healthcare Life Sciences), 1% penicillin-streptomycin (1000 U/ml) (#SV30010, Hyclone, GE Healthcare Life Sciences), and 1% amphotericin B (25 mg/ml) (#SV30078.01, Hyclone, GE Healthcare Life Sciences), at 37ºC in a 5% CO_2_ atmosphere. SKO, Staufen1 knockout cells were generated from parental HCT116 cells as previously described (36,60), and both cell lines were maintained in McCoy's Media (#16600082 Life Technologies, Thermo Fisher Scientific Inc.) supplemented with 1% penicillin/streptomycin and 10% fetal bovine serum (Hyclone).

### DNA transfection

HEK 293T, SKO, and HCT116 cells were seeded at 1.1 × 10^5^ cells per well in 24-well culture plates. DNA transfection experiments were performed at 60–70% confluency using polyethyleneimine (PEI; GIBCO, ThermoFisher Scientific Inc). The cells were cotransfected with 300 ng of dl-plasmids, together with the indicated concentration (ng) of Stau1^55^-TAG plasmids or plasmid pSP64 Poly(A) (#P1241, Promega Corporation, Madison, WI, USA); the last was used as a filler DNA to keep the final DNA concentration constant in all transfection assays. In all experiments 24 h after transfection (unless indicated otherwise in the text), the culture medium was removed, and the cells were harvested using Passive Lysis Buffer supplied with the Dual-Luciferase® Reporter Assay System (#E1960, Promega Corporation) according to manufacturer's protocols. HeLa cells were seeded onto sterile coverslips (18 mm øNo. 1 German cover glasses, VWR VistaVision™, VWR International, Radnor, Pennsylvania, USA) deposited into 12-well plates. Cells were transfected 24 h later with 2 μg pNL-4.3 plasmid DNA per well using JetPrime (PolyPlus, Illkirch-Graffenstaden, Francia) according to the manufacturer's instructions. Cells were fixed 24 h later.

### siRNA-DNA co-transfection

HEK 293T cells were seeded at 1.2 × 10^5^ cells per well in 24-well culture plates. Endogenous Staufen1 protein silencing was performed over 70–80% confluent cells using the Lipofectamine 2000 system (#11668019, Invitrogen, ThermoFisher Scientific Inc). For Staufen1 silencing, a duplex siRNA 3084 (si55/63) (5′-AAATAGCACAGTTTGGAAACT-3′; Integrated DNA Technologies, IDT, Coralville, IA, USA) previously described (32), targeting Staufen1 was used at 50 nM for 48h, together with 200 ng of dl HIV-1 IRES or of the pNL-4.3-RLuc plasmid. For RLuc silencing, 100 nM of a duplex siRNA targeting the RLuc open reading frame (Renilla siRNA 2, 5′-UAUAAGAACCAUUACCAGAUUUGCCUG-3′, Integrated DNA Technologies, IDT) (24,61,62) was cotransfected with 150 ng of dl-plasmid and pSP64 Poly(A) or Stau1^55^-HA_3_ plasmid for 48 h. The Silencer Select Negative Control #1 siRNA (#4390844, Ambion, Thermo-Fisher Scientific Inc.), was used as a non-related scrambled RNA (scRNA) negative control.

### Luciferase assays

The activities of Firefly luciferase (FLuc) and *Renilla* luciferase (RLuc) were measured using the DLR® Assay System (#E1960, Promega Corporation) according to the manufacturer's instructions, on 10 μl of cell lysates using a Sirius Single Tube Luminometer (Berthold Detection Systems GmbH). Data are expressed as a percentage of the Relative Luciferase Activity (RLA) or as Relative Translation Activity (RTA); the latter corresponding to the FLuc/RLuc ratio, an index of the IRES activity (16,18,24,61–63).

### RNA extraction and real-time RT-qPCR

Cytoplasmic RNA from HEK 293T cells was extracted using the protocol described (64). Cells were washed twice with PBS 1X (#SH30256, Hyclone, GE Healthcare Life Sciences), incubated 5 min on ice with RNLa buffer (10mM Tris–HCl pH 8, 10 mM NaCl, 3 mM MgCl_2_, 0.5% NP-40, 1 mM DTT) containing 10 U of Ribolock RNase inhibitor (#EO0381, Thermo-Fisher Scientific Inc.). After incubation 500 μl of TRIzol reagent (#15596018, Life Technologies Corporation, Thermo Fisher Scientific Inc.) was added to the supernatant, and the RNA was recovered. Cytoplasmic RNA was resuspended in 20 μl of nuclease-free water, DNase-treated (#AM1907, Ambion, Thermo-Fisher Scientific Inc.), and recovered according to the manufacturer's instructions. The RNA concentration was quantified by nano-spectrophotometry (N60-Implen Nanophotometer, Westlake Village, CA, USA). The real-time RT-qPCR experiments were carried out using the Brilliant II SYBR Green RT-qPCR one Step Master Mix (#600835, Agilent Technologies, Santa Clara, CA, USA). Gag-RLuc RNA was amplified using primers Renilla sense (5′-AGGTGAAGTTCGTCGTCCAACATTATC-3′) and Renilla antisense (5′- GAAACTTCTTGGCACCTTCAACAATAGC-3′) as previously described (24). No-RT-qPCR reactions were carried out to control for contaminant DNA. GAPDH mRNA was detected with the primers GAPDH sense (5′-TCCACCACCCTGTTGCTGTAG-3′) and GAPDH antisense (5′-ACCCACTCCTCCACCTTTGAC-3′) as previously described (65). Data analysis was performed by the ΔΔCt method as previously described in (66).

### Cell viability and proliferation assays

The cell viability assay was performed using the CellTiter 96® Aqueous One Solution Cell Proliferation Assay (MTS) (#G358A, Promega Corporation) according to the manufacturer's instructions. Briefly, HEK 293T cells were seeded at 1.5 × 10^3^ cells per well in a 96-well plate and transfected with the indicated concentrations of sc/siRNAs or transfected with the indicated amounts of Stau1^55^-HA_3_ expressing plasmid. 24 or 48 h later (as indicated in the text), the CellTiter 96^®^ Aqueous One Solution Cell Proliferation Assay was added, incubated at 37ºC for 2 h, and the absorbance was measured at 495nm in a Biochrom EZ Read 400 microplate reader (Biochrom, Holliston, MA, USA). Alternatively, cell viability and cell proliferation were analyzed by flow cytometry (FC) (67). HEK 293T, HCT116 or SKO cells were seeded in 24 well-plate and transfected with the Stau1^55^-HA_3_ (200 or 650 ng) expressing plasmid. After 48h of incubation, cells were incubated in human Fc block (#564220 BD Biosciences, San Jose, CA, USA) and Fixed Viability Stain 510 (#564406, BD Biosciences) for 15 min at room temperature, followed by an incubation of 20 min in fixation and permeabilization solution (#554722, BD Biosciences), and incubated with the BUV395 mouse anti-Ki67 antibody (#564071, BD Biosciences) for 30 min at room temperature. The single stain control was prepared using Compensation Beads (#552843, BD Biosciences). An average of 20,000 cells stained for FC was acquired using a BD LSRFortessa (BD Biosciences) at the Flow Cytometry Core Facility, Lady Davis Research Institute. Data were analyzed using FlowJo software (Tree Star).

### Western blots

Cells were lysed using the Passive Lysis 5× Buffer (#E1941, Promega Corporation) or NP40 lysis buffer (50 mM Tris pH 7.4, 150 mM NaCl, 0.5 mM EDTA, 0.5% NP40). The concentration of total protein was determined by the Bradford assay using the Bio-Rad Protein Assay (#5000006, Bio-Rad Laboratories, Inc., Hercules, CA, USA). Equal amounts of protein (20 or 30 μg) were resolved by electrophoresis on a 12% glycine sodium dodecyl sulfate polyacrylamide gel (SDS-PAGE) or 15% SDS-PAGE and transferred onto a 0.2 (# 1620112, Bio-Rad Laboratories) or 0.45 μm nitrocellulose membrane (#10600002; GE Healthcare Bio-Sciences 100 Results Way, Marlborough, MA, USA). Membranes were blocked with Tris-buffered saline (pH7.4) containing 5% skimmed milk and 0.1% Tween-20 (TBST) for 1 h at room temperature, washed three times with TBST, and incubated overnight at 4ºC with the primary antibody. The membranes were then washed three times with TBST and incubated with the corresponding horseradish peroxidase-conjugated secondary antibodies The primary rabbit anti-Staufen1 antibody (#175) was generated at the McGill University Cell Imaging and Analysis Network (https://www.mcgill.ca/cian/) using recombinant full-length Staufen1 as antigen and used in western blotting analyses blotting buffer at 1:1000 dilution, a primary rabbit antibody was used for combined immunofluorescence/fluorescence *in situ* hybridization analyses was described previously (31,68) a mouse anti-HA (#H9658, Sigma-Aldrich, 3050 Spruce Street, St. Louis, MO, USA) was used at a 1:5000 dilution, a rabbit anti-GFP (# NB600-308, Novus Biologicals, Toronto, ON, Canada) at a 1:5000 dilution, a mouse anti-GAPDH (#MA5-15738; Thermo Fisher Scientific Inc.) at a 1:5000 dilution (loading control), a rabbit anti-β Actin (#ab8227; Abcam Inc, Toronto, ON, Canada) at a 1:5000 dilution, a rabbit anti-p17 HIV-1 (#4811, NIH AIDS Reference and Reagents program; kindly shared by Dr R. Soto-Rifo, Programa de Virología, ICBM, Universidad de Chile) at a 1:1000 dilution. Either a Goat anti-mouse or Goat anti-rabbit IgG-horseradish peroxidase (HRP) conjugate (#AP308P, #AP132P; Merck, Darmstadt, Germany) secondary antibodies, both at 1:10,000 dilution, were used. Western blots were visualized by enhanced luminescence by a chemiluminescence reaction using 4-hydroxycinnamic acid (#800237, Merck) and luminol (#09253, Sigma-Aldrich), the SuperSignal™ West Femto Maximum Sensitivity Substrate (#34096, Thermo Fisher Scientific Inc.) or Western Lightning Plus-ECL (# NEL 121001, PerkinElmer Health Science Canada, Inc, Ontario, Canada). The western blot films (Fuji medical X-ray film Super HR-U 30 or Hyblot CL (# DV-3012, Denville Scientific Inc., NJ, USA)) were digitized using a CanonScan 9950F scanner, or membrane chemiluminescence was captured using an Alliance 2.7 imaging system (UVItec Cambridge, Topac Inc., 231 CJC Highway, Cohasset, MA, USA) as in (69).

### Fluorescence in situ hybridization, immunofluorescence, and microscopy

**Figure 1.**
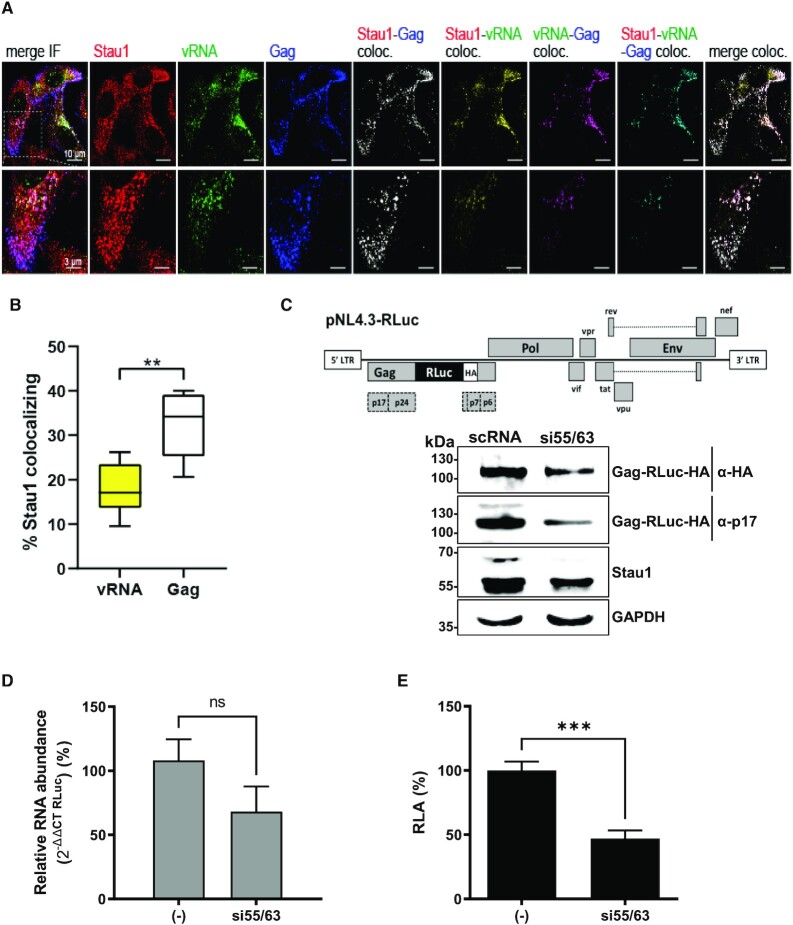
Staufen1 binds the vRNA and regulated HIV-1 Gag expression. (**A**) HeLa cells were transfected with pNL-4.3 proviral DNA and at 48 h post-transfection, combined IF/FISH was performed as described in Materials and Methods for endogenous Staufen1 (red), HIV-1 vRNA (green), and HIV-1 Gag (blue). A merged panel showing colocalization Staufen1, HIV-1 vRNA and HIV-1 Gag signals is shown on the far left (last panel), while resulting colocalization channels between Staufen1-Gag (white), Staufen1-vRNA (yellow), vRNA-Gag (magenta), and Staufen1-vRNA-Gag (cyan), as determined using Imaris software, are shown, as indicated. (**B**) Imaris was used to determine percent colocalization between Staufen1 and vRNA (17.70% ± 5.79 S.D.; yellow bar) and Staufen1 and Gag (32.07% ±7.38 S.D., white bar). Statistical analyses were performed by an unpaired two-tailed *t-test* (*P* = 0.0016). (**C**) Schematic representation of the complete HIV-1 molecular clone pNL-4.3-RLuc (upper panel). HEK 293T cells were cotransfected with the pNL-4.3-RLuc (200 ng) plasmid and 50 nM of a duplex siRNA targeting the Staufen1 mRNA (si55/63), or with a scrambled RNA ((−); 50 nM) as a control. The expression of the HIV-1 Gag-RLuc-HA fusion protein and endogenous Staufen1 was determined 48 h post-transfection by western blot, using the GAPDH protein as a loading control. (**D**) Cytoplasmic RNA was extracted from cells expressing pNL-4.3-RLuc HIV-1 and treated with the scRNA or the si55/63 (50 nM), and relative RNA levels were determined by real-time RT-qPCR. The RNA abundance was expressed relative to the value obtained for the cells treated with the scRNA set to 100%. (**E**) *Renilla* luciferase activity was measured 48 h post-transfection and is expressed as relative luciferase activity (RLA) relative to the activity obtained when the pNL-4.3-RLuc plasmid was cotransfected with the scRNA (−). In (**D**) and (**E**) values represent the mean (±SEM) for seven independent experiments, each conducted in duplicate. Statistical analyses were performed by an unpaired two-tailed *t-test* (* *P* < 0.01; *** *P* < 0.0005).

Fluorescence *in situ* hybridization (FISH) and immunofluorescence (IF) analyses on HeLa cells were performed as described previously (36,70). To fix cells onto cover glasses, cells were washed once in D-PBS (Wisent) and fixed with 4% paraformaldehyde for 20 min. Fixed cells were then washed with D-PBS, quenched in 0.1 M glycine for 10 min, washed with D-PBS, permeabilized in 0.2% Triton X-100 for 5 min, and washed twice with D-PBS. For FISH/IF co-analyses (Figure 1A), a digoxigenin (DIG)-labeled RNA probe was synthesized *in vitro* in the presence of DIG-labeled UTP (Roche). For FISH probe hybridization to stain the vRNA, cells were DNase (Invitrogen) treated for 15 min (25 U per coverslip), then incubated in hybridization solution for 16–18 h at 42°C (50% formamide, 1 mg/ml tRNA, 2× SSPE, 5× Denharts, 5 U RNaseOut (Invitrogen), 50 ng probe). Cells were then incubated in 50% formamide for 15 min at 42°C and incubated twice in 2× SSPE for 5 min each at 42°C. Cells were briefly washed in PBS before being blocked in 1× blocking solution (Roche). Primary antibodies (mouse anti-p24, NIH AIDS ARRP; rabbit anti-Staufen1 (Graciela Boccaccio, Instituto Leloir Buenos Aires, Argentina) (31,68,71); sheep anti-DIG-AP, Fab fragments, Roche #11093274910) were applied at 1:250 for 1 h at 37°C, and then washed for 10 min in PBS followed by secondary antibodies (Donkey anti-Sheep IgG (H + L) Cross-Adsorbed Alexa Fluor® 488 #A-11015; Donkey anti-Rabbit IgG (H + L) Highly Cross-Adsorbed, Alexa Fluor^®^ 594 #A-21207, and Donkey anti-Mouse IgG (H + L) Highly Cross-Adsorbed, Alexa Fluor^®^ 647 #A-31571, by Invitrogen-Thermo Fisher Scientific) were applied at 1:500 for 1 h at 37°C. Coverslips were washed for 20 min in PBS before being mounted on glass slides using ProLong Gold Antifade Reagent (Life Technologies). Microscopy was performed on a Zeiss LSM5 Pascal laser-scanning confocal microscope (Carl-Zeiss) equipped with a 63× (1.4 numerical aperture, oil immersion) plan-apochromat objective. Scanning was performed at 1024 × 1024 pixel resolution using a multitrack laser scanning protocol. Image files into Imaris software v. 9.7 for generation of colocalization channels. For IF analysis performed in Figure 6, cells were fixed, permeabilized, and blocked as described above, and then incubated with primary antibodies (sheep anti-GFP, Novus Biologicals, #NB100-62622; mouse anti-(Firefly) Luciferase, Sigma-Aldrich, # L2164; rabbit anti-Renilla Luciferase, MBL, #PM047) applied at 1:250 for 1 h at 37°C, and then washed for 10 min in PBS followed by secondary antibodies (Donkey anti-Sheep IgG (H + L) Cross-Adsorbed Alexa Fluor® 488 #A-11015; Donkey anti-Mouse IgG (H + L) Highly Cross-Adsorbed, Alexa Fluor® 594 #A-21203; Donkey anti-Rabbit IgG (H + L) Highly Cross-Adsorbed, Alexa Fluor^®^ 647 #A-31573; Invitrogen-Thermo Fisher Scientific) were applied at 1:500 for 1 h at 37°C. Coverslips were washed for 20 min in PBS before being mounted on glass slides using ProLong Gold Antifade Reagent with DAPI (Life Technologi.es). Laser confocal microscopy was performed using a Leica DM16000B microscope equipped with a WaveFX spinning disk confocal head (Quorum Technologies) and HCX PL APO/40×, oil/0.75–1.25 NA CS and HCX PL APO/63×, oil/0.60–1.40 NA BL objectives, and images were acquired with a Hamamatsu EM-charge coupled digital device camera. Scanning was performed at a thickness of 1 μm and at a resolution of 1024 × 1024 pixels. For multi-color image capture, AlexaFluor-647, 594, 488, conjugated secondary antibody emissions were sequentially captured with 665–715, 570–620 and 500–550, bandpass filters, followed by 435–485 nm [for 4′,6-diamidino-2-phenylindole (DAPI) staining]. Raw .liff files were exported by the Volocity software (Perkin Elmer) for import into Imaris and ImarisColoc software v. 9.7 (Bitplane/Andor), and .csv exports of quantitative measurements of mean signal intensity (MFI) values used for statistical analyses by Prism v6.1 (GraphPad, 2365 Northside Dr., San Diego, CA, USA).

### Statistical analysis

Graphics and statistical analysis were carried out using the Prism v6.1 or v9.2.0 software (GraphPad), performing the statistical test indicated in the text and figure legends.

## RESULTS

### Endogenous Staufen1 interacts with the HIV-1 vRNA and regulates viral protein synthesis

Previous reports show that in cells replicating HIV-1 endogenous Staufen1 associates with the vRNA and with newly synthesized Gag protein (31,32,35,36,53,72). As a starting point for this study, we sought to confirm the Staufen1-vRNA association in cells replicating HIV-1. For this, HeLa cells were transfected with the HIV-1 proviral DNA (pNL-4.3) (56). The localization of endogenous Staufen1 in relationship to the HIV-1 vRNA and Gag was examined *in situ* by using combined immunofluorescence and fluorescence *in situ* hybridization analysis (IF/FISH) as previously described (31,68). In accordance with previous reports (31,32,34,35), which show reciprocal immunoprecipitation assays between Staufen1 and HIV-1 Gag (31), the results confirm that in HIV-1-expressing HeLa cells, Staufen1, vRNA, and Gag exhibited colocalization (Figure 1A and B), validating the close association of Staufen1 with viral components in cells. Importantly, Staufen1 colocalized with the vRNA (Figure 1A (merge IF and Staufen1-vRNA) and 1B). Interestingly, Staufen1-vRNA, Staufen1-Gag and vRNA-Gag colocalizations did not entirely overlap (Figure 1A, merge IF). In accordance with previous reports suggesting the formation of different Saufen1-Gag and Saufen-vRNA complexes in HIV-1-expressing cells (31,32,34,52), results show an uneven percentage of colocalization between Staufen1 and vRNA ($\sim$18%) and Staufen1 and Gag ($\sim$32%) (Figure 1B).

Next, we sought to determine if Staufen1 participates in Gag protein synthesis. For this, HEK 293T cell lines were transfected with the pNL-4.3-RLuc DNA (Figure 1C, upper panel), which has a hemagglutinin (HA)-tagged Renilla luciferase (RLuc-HA) reporter gene inserted in frame with the Gag-protein start codon, generating a Gag-RLuc-HA fusion protein (57). This plasmid allows a direct evaluation of Gag synthesis from the HIV-1 vRNA, using the Gag-RLuc-HA reporter and its luciferase activity as a readout (25,57,69,73). HEK 293T cells were cotransfected with the pNL-4.3-RLuc plasmid and a short interfering RNA (siRNA) si55/63 (50 nM) targeting Staufen1 mRNAs (31,32) or a non-related scrambled control siRNA (scRNA; 50 nM). In agreement with earlier reports (31,33,55,74,75), when using the anti-Staufen1 antibody three bands were observed (Figure 1C), one band for the 63 kDa isoform and a doublet, associated with an upstream AUG in the N-terminal end of Stau1^55^ that enables alternative translation initiation (38), for the 55 kDa isoform. As expected (31,32), treatment of cells with si55/63 reduced the expression of endogenous Staufen1 (Figure 1C, lower panel). A reduction in the Gag-RLuc-HA expression levels was detected in cells treated with the si55/63 by either using an antibody against the matrix (p17) region of Gag or the HA-tag (Figure 1C, lower panel). Cytoplasmic RNA was extracted from pNL-4.3-RLuc HIV-1, scRNA, or si55/63 cotransfected cells and used as a template for quantitative analysis of the pNL-4.3RLuc RNA by an RT-qPCR. The relative pNL-4.3-RLuc mRNA content was reduced ($\sim$30% reduction), however, not significantly in si55/63 treated cells, suggesting that the resulting decrease in Gag-RLuc-HA levels (Figure 1C) was not exclusively associated with a reduction in the abundance of HIV-1 vRNA (Figure 1D). We also determined the luciferase activity of the fusion protein. Consistent with a decrease in protein levels (Figure 1C), a significant (*P* < 0.05) decrease in RLuc activity ($\sim$53% reduction) in cells treated with the si55/63 was also observed (Figure 1E). These results confirm previous reports (31–33,35) and show that Staufen1 contributes to HIV-1 vRNA translation.

### Staufen1 participates in HIV-1 IRES-mediated translation in cells

**Figure 2.**
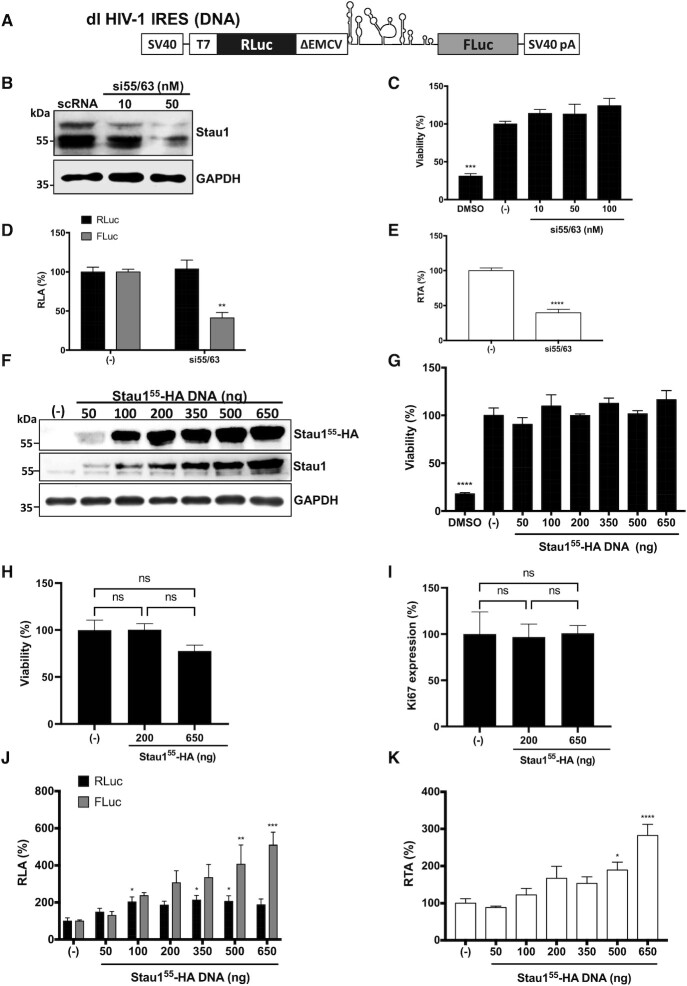
Staufen1 is an ITAF for the HIV-1 IRES. (**A**) Schematic representation of dl HIV-1 IRES that harbors the 5′UTR of the HIV-1 mRNA (pNL-4.3 HIV-1 clone, GenBank accession number AF 324493) in the intercistronic spacer flanked by *RLuc* and *FLuc* ORFs (16). (**B–E**) The dl HIV-1 IRES plasmid (300 ng) was cotransfected with the si55/63 targeting *Staufen* mRNA (10–50 nM) or with the scRNA ((−); 50 nM) in HEK 293T cells. (**B**) The expression level of endogenous Stau1^55^ and Stau1^63^ was determined by western blot using an anti-Staufen1 antibody and GAPDH as a loading control. (**C**) Cell viability was determined by measuring the cellular metabolic activity using the MTS assay using dimethylsulfoxide (DMSO, 10%) as a control for cell death. Data are expressed relative to the viability of the cells transfected with scRNA(−) set to 100%. Values shown are the mean (±SEM) for three independent experiments, each performed in duplicate. Statistical analysis was performed by an ordinary one-way ANOVA test (***P < 0.001). (**D, E**) RLuc and Fluc activities were measured 48 h post-transfection, and data are presented as RLA (**D**) and relative translational activity (RTA) (**E**), with the values obtained in the presence of the scRNA (−) set to 100%. RTA corresponds to the [FLuc/RLuc] ratio that is used as an index of IRES activity. Values represent the mean (±SEM) for three independent experiments, each conducted in duplicate. Statistical analysis was performed by an ordinary two-way ANOVA test (** *P* < 0.005, **** *P* < 0.0001). (**F**–**K**) HEK 293T cells were cotransfected with the dl HIV-1 IRES (300 ng) and different quantities (50–650 ng) of a plasmid encoding for a Stau1^55^-HA_3_ protein. The expression of Stau1^55^-HA_3_ was confirmed by western blot using an anti-HA or anti-Staufen1 antibody and GAPDH as a loading control. (**G, H**) The viability of HEK 293T cells was determined either by measuring the cellular metabolic activity using the MTS assay and dimethylsulfoxide (DMSO, 10%) as a control for cell death (**G**) or by using flow cytometry (FC) in cells stained with Fixed Viability Stain 510 and determining the (5) of living cells (**H**). In (**G, H**), data are expressed relative to the viability of the cells transfected with the negative control set to 100%. (I) Cell proliferation was analyzed by detecting Ki67 protein in HEK 293T cells transfected or not with the Stau1^55^-HA_3_ expressing plasmid (200 or 650 ng). For (**G–I**) values are the mean (±SEM) for three independent experiments, each performed in duplicate. Statistical analysis was performed by an ordinary one-way ANOVA test (**P < 0.005, ****P < 0.0001; ns, not significant). (**J, K**). RLuc and FLuc activities were measured 24 h post-transfection, and data are presented RLA (**J**) or RTA (**K**). The RLA and RTA values obtained in the absence (−) of the Stau1^55^-HA_3_ plasmid were set to 100%. Values shown are the mean (±SEM) of four independent experiments, each performed in duplicate. Statistical analysis was performed by an ordinary one-way ANOVA test (* *P* < 0.05, ** *P* < 0.005, ***P < 0.001, ****P < 0.0001).

Stau1^55^ colocalizes with the HIV-1 vRNA in the cytoplasm and plays a role in its translation ((31–33,35) and Figure 1). However, Gag-RLuc-HA fusion protein expression from the pNL-4.3-RLuc HIV-1 clone (Figure 1) does not allow the discrimination between cap-dependent or IRES-mediated translation initiation (8,9). Knowing that Stau1^55^ regulates cap-dependent translation (33), we next focused exclusively on determining if Staufen1 was important for HIV-1 IRES-activity. For this, we used a dual-luciferase (dl) reporter plasmid, dl HIV-1 IRES (16,19,24,27,69). The dl HIV-1 IRES DNA encodes a bicistronic capped and polyadenylated mRNA with an upstream *Renilla* luciferase (RLuc) open reading frame (ORF), followed by a deleted 5′UTR of the *encephalomyocarditis virus* (ΔEMCV), the 5′UTR (nucleotides 1–336) of the vRNA of pNL-4.3, and firefly luciferase (FLuc) ORF ((16) and Figure 2A). The highly structured ΔEMCV sequence, deficient in IRES activity, impedes ribosome reinitiation and readthrough (16,18,76). This well-characterized dl-RNA has proven to be a useful molecular tool to study HIV-1 IRES function (16,18,19,22,24–28,69). First, we examined whether the reduction of endogenous Staufen1 influenced HIV-1 IRES-mediated translation in cells. HEK 293T cells were chosen for this experiment because they are readily transfectable, and have been used as a model system to study HIV-1 replication (77), HIV-1 IRES activity (69), and the impact of Staufen1 on HIV-1 replication (32,35,72). Thus, HEK 293T cells were cotransfected with the dl HIV-1 IRES plasmid and a scRNA control, or different concentrations of the si55/63 (31,32). Treatment of cells with the si55/63 (50 nM) led to a marked reduction in Staufen1 (Figure 2B) with no associated effect on cell viability (Figure 2C). Luciferase activities were measured, and data were expressed as relative luciferase activity (RLA). The expression of RLuc and FLuc obtained from cells transfected with the scRNA(−) was set to 100% (Figure 2D). A significant decrease ($\sim$60%) of HIV-1 IRES activity (FLuc) with no impact on cap-dependent translation (RLuc) was evidenced in cells transfected with the dl HIV-1 IRES plasmid treated with the si55/63 (50 nM) (Figure 2D). As Staufen1 knockdown did not significantly alter RLuc activity, the observed reduction in FLuc activity cannot be attributed to reduced stability of the dl HIV-1 IRES mRNA (Figure 2D). Analysis of the FLuc/RLuc ratio (relative translational activity, RTA), as an index of IRES activity (16,18,24), confirmed the significant reduction ($\sim$60%) in HIV-1 IRES activity when the endogenous Staufen1 protein was depleted (Figure 2E), recapitulating what was observed with the pNL-4.3-RLuc mRNA (Figure 1B–D). Thus, this observation confirms that Staufen1 contributes to HIV-1 IRES-mediated translation in HEK 293T cells

Next, we sought to evaluate the impact of Stau1^55^ on HIV-1 IRES activity. For this, HEK 293T cells were cotransfected with the dlHIV-1 IRES plasmid, an irrelevant DNA (negative control), or different concentrations (50–650 ng) of plasmid Stau1^55^-HA_3_, encoding for a hemagglutinin (HA_3_)-tagged Stau1^55^ (47). The overexpression of Stau1^55^-HA_3_, confirmed by western blot using GAPDH as a loading control (Figure 2F). As previously described, Stau1^55^-HA_3_ migrated at the level of Stau1^63^ when resolved by electrophoresis (33,55). The overexpression of Stau1^55^-HA_3_ has been reported to impair cell viability and proliferation (46,55,74). However, in agreement with a previous report using asynchronous HEK 293T cells (55), in the time frame of the experiment (24–48 h), the overexpression Stau1^55^-HA_3_ did not impair cell viability (Figure 2G, H) or cell proliferation (Figure 2I). Luciferase activities were measured, and data expressed as RLA, with the values of RLuc and FLuc obtained from cells transfected with the control DNA(−) set to 100% (Figure 2J). At lower levels of Stau1^55^-HA_3_ expression (50–100 ng of DNA), RLuc and FLuc increased equivalently (Figure 2J), most probably due to RNA stabilization (33,48). Proteins involved in mRNA stability are known to associate with Stau1^55^ (34). However, as the levels of recombinant Stau1^55^-HA_3_ increased (200–650 ng of DNA), FLuc activity was enhanced (to a maximum of $\sim$5-fold) without having a further significant impact on the expression RLuc activity (Figure 2J). Analysis of the FLuc/RLuc ratio (RTA), better illustrates the concentration-dependent increase and significantly higher activity of the HIV-1 IRES when the Stau1^55^-HA_3_ protein is overexpressed (500 and 650 ng of DNA; Figure 2K). These results suggest that Stau1^55^ promotes HIV-1 IRES activity.

### Overexpression of Staufen1 does not induce alternative splicing nor cryptic promoter activity from the dl HIV-1 IRES reporter in cells

**Figure 3.**
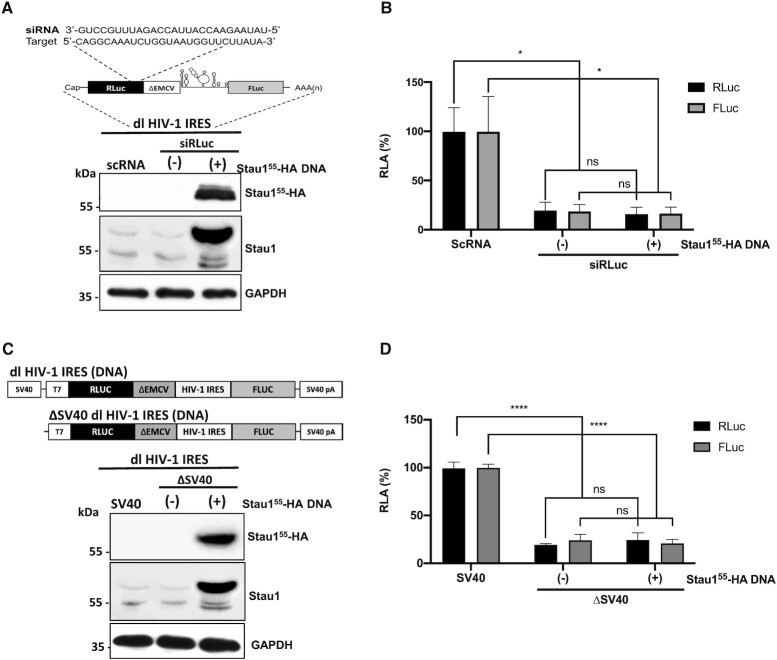
Staufen1 does not enhance alternative splicing of the dl HIV-1 IRES RNA nor increases the cryptic promoter activity of the dl HIV-1 DNA. (**A**, **B**) The dl HIV-1 IRES (150 ng) was cotransfected with a control scRNA (100 nM) or with siRLuc (100 nM), in the presence, or the absence (−), of the Stau1^55^-HA_3_ (325 ng) plasmid. (**A**) Schematic representation of the dl reporter targeted by the siRNA RLuc (siRLuc) targeting the *Renilla* luciferase ORF (upper panel). Total protein extracts were prepared 48 hrs post-transfection. The expression of Stau1^55^-HA_3_ was determined by western blot, using the GAPDH protein as a loading control (lower panel). (**B**) RLuc and FLuc activities were measured and expressed relative to the values obtained with scRNA, set to 100% (RLA). Values shown are the mean (±SEM) for six independent experiments, each performed in duplicate. Statistical analysis was performed by an ordinary two-way ANOVA test (*P < 0.01; ns, not significant). (**C**, **D**) HEK 293T cells were transfected with either the dl HIV-1 IRES (150 ng) or a promoterless ΔSV40-dl HIV-1 IRES (150 ng) vector in the presence, or the absence (−), of the Stau1^55^-HA_3_ (325 ng) plasmid. 24 hrs post-transfection total protein extracts were prepared. (**C**) Schematic representation of the dl HIV-1 IRES and ΔSV40-dl HIV-1 IRES plasmids (upper panel). The expression of Stau1^55^-HA_3_ was determined by western blot, using the GAPDH protein as a loading control (lower panel). (**D**) RLuc and FLuc activities were measured, and results are expressed as RLA relative to the activities obtained from the dl HIV-1 IRES vector when in the absence of the Stau1^55^-HA_3_, set to 100%. Values shown in are the mean (±SEM) for three independent experiments, each performed in duplicate. Statistical analysis was performed by an ordinary two-way ANOVA test (**** *P* < 0.0001; ns, not significant).

Stau1^55^ interacts with splicing factors and is involved in pre-mRNA splicing (34,78). Therefore, Stau1^55^-HA_3_ overexpression could generate a monocistronic mRNA encoding a functional FLuc protein from the dl HIV-1 IRES RNA by inducing an alternative splicing event (79). If so, the increase in FLuc activity (Figure 2J) would not reflect HIV-1 IRES activity but would correspond to the expression of a cap-dependent monocistronic FLuc encoding mRNA. To explore this possibility, the *Renilla* ORF of the dl HIV-1 IRES mRNA was targeted with an RLuc-siRNA (siRLuc) (Figure 3A, upper panel), as previously described (24,69). The rationale for this experiment considers that if a monocistronic transcript encoding an active FLuc enzyme is indeed generated from a cryptic promoter or through RNA splicing, using a short interfering RNA (siRNA) that targets the RLuc coding region should knockdown the bicistronic RNA without affecting the expression levels of a monocistronic *FLuc* transcript. The siRLuc RNA or a scRNA were cotransfected with the dl HIV-1 IRES in HEK 293T cells. The expression of Stau1^55^-HA_3_ was confirmed by western blot using an anti-HA or anti-Staufen1 antibody and GAPDH as a loading control (Figure 3A, lower panel). In the presence of the siRLuc RNA, both RLuc and FLuc activities were significantly reduced, whether Stau1^55^-HA_3_ was overexpressed or not (Figure 3B). When directly compared, the reduction of RLuc and FLuc activities induced by the siRLuc RNA in the presence, or in the absence, of overexpressed Stau1^55^-HA_3_ protein was not statistically different (Figure 3B). This observation indicated that RLuc and FLuc expression levels were associated with a single transcript targeted by the siRLuc RNA and confirmed that the overexpression of Stau1^55^ does not induce the generation of FLuc expressing monocistronic transcripts in HEK 293T cells.

The dl HIV-1 IRES reporter plasmid displays cryptic promoter activity in HEK 293T cells (69). In addition, the FLuc reporter gene also exhibits cryptic promoter activity that is detectable in both yeast and mammalian cells (80). However, the identified promoter lies within the FLuc coding sequence generating short mRNAs that do not code for a functional luciferase enzyme (80). Based on these previous reports, we next sought to directly assess if the overexpression of Stau1^55^ enhanced cryptic promoter activity present within the used dl-plasmid in HEK293T cells. For this, cells were transfected with the dl HIV-1 IRES or the ΔSV40 dl HIV-1 IRES plasmid in the presence or absence of the Stau1^55^-HA_3_ expressing plasmid. The ΔSV40 dl HIV-1 IRES plasmid lacks the SV40 promoter (Figure 3C, upper panel) (19,24,69). The expression of the Stau1^55^-HA_3_ was confirmed by western blot analysis using an anti-HA or anti-Staufen1 antibody and GAPDH as a loading control (Figure 3C, lower panel). In the absence of the SV40 promoter (ΔSV40), both the RLuc and FLuc activities from the reporters were significantly (*P* < 0.05) diminished in cells (Figure 3D). However, in agreement with a previous report (69), RLuc activity was detected in HEK 293T, suggesting a leakiness of about 19% existed (Figure 3D). Our results also showed that FLuc activity was 9% higher than RLuc, confirming the presence of weak cryptic promoter activity in HEK 293T (69). The overexpression of Stau1^55^-HA_3_ did not impact FLuc expression from the ΔSV40 dl HIV-1 IRES plasmid, indicating that Stau1^55^-HA_3_ does not enhance cryptic promoter activity from the used vector in HEK 293T cells (Figure 3D).

Based on the above results, we conclude that overexpression of Stau1^55^-HA_3_ does not induce the expression of a monocistronic mRNA encoding for an active FLuc enzyme by enhancing splicing (Figure 3A and B) or by increasing cryptic promoter activity (Figure 3C and D) from the dlHIV-1 IRES reporter in HEK 293T cells. Thus, results in Figure 2 reflect the function of a genuine IRES and indicate that Stau1^55^ promotes HIV-1 IRES activity in HEK 293T cells.

### The TAR and poly(A) Stem loops of the HIV-1 5′UTR participate in Staufen1 stimulation of HIV-1 IRES activity in cells

**Figure 4.**
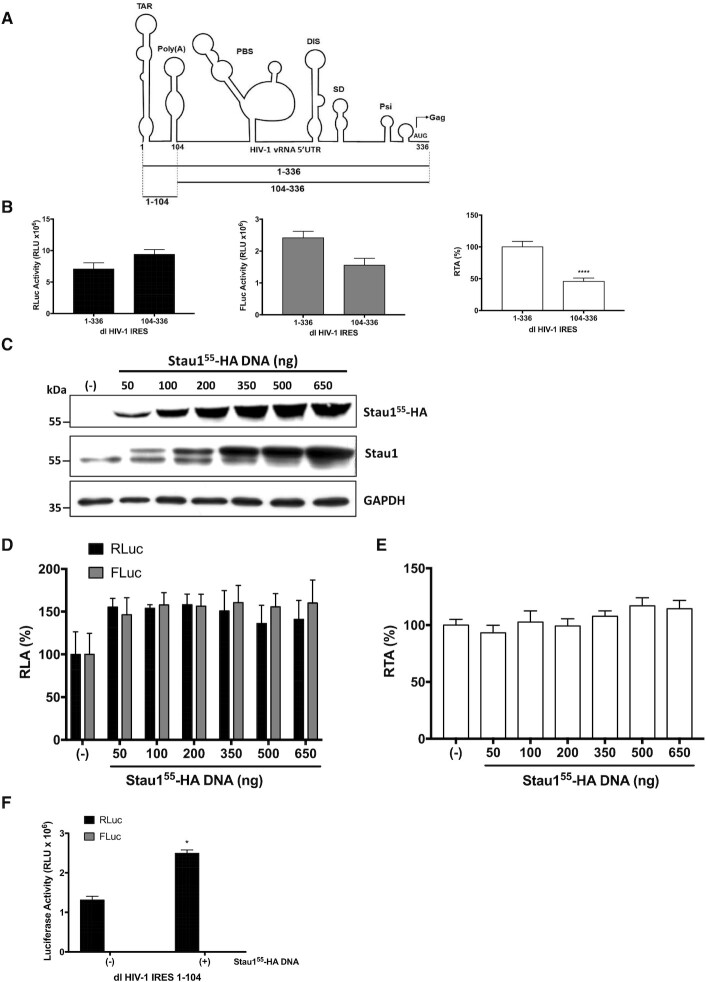
Overexpression of Staufen1 does not stimulate the activity of a 5′deletion mutant of the HIV-1 IRES lacking the TAR and Poly(A) stem-loops. (**A**) Schematic representation of the pNL-4.3 vRNA 5′UTR used to generate dl HIV-1 plasmids (intercistronic region), dl HIV-1 IRES (nts 1–336), dl HIV-1 IRES 104–336 (nts 104–336), and dl HIV-1 IRES 1–104 (nts 1–104). The different RNA structural elements (TAR, poly(A) loop, PBS, DIS, SD and Psi) present within the HIV-1 vRNA 5′UTR are depicted (21). The arrow at position 336 indicated the Gag ORF initiation codon. (**B**) HEK 293T cells were transfected with the dl HIV-1 IRES or dl HIV-1 IRES 104–336 (300 ng) plasmids. RLuc (left panel) and FLuc (middle panel) activities were measured 24 h post-transfection, and data are presented as relative light units (RLU) or as RTA (right panel). (**C−E**) HEK 293T cells were cotransfected with the dl HIV-1 IRES 104–336 (300 ng) and different quantities (50–650 ng) of a plasmid encoding for a Stau1^55^-HA_3_. (**C**) The presence of the overexpressed Stau1^55^-HA_3_ was confirmed by western blot using an anti-HA or anti-Satufen1 antibody and GAPDH as a loading control. (**D, E**) RLuc and FLuc activities were measured 24 h post-transfection, and data are presented as RLA (**D**) or RTA (**E**). The RLA and RTA values obtained in the absence (−) of the Stau1^55^-HA plasmid were set to 100%. Values shown are the mean (+/-SEM) of four independent experiments, each performed in duplicate. Statistical analysis was performed by an ordinary one-way ANOVA test. (**F**) HEK 293T cells were cotransfected with the dl HIV-1 IRES 1–104 (300 ng) and 650 ng of a plasmid encoding for a Stau1^55^-HA_3_ protein. RLuc and FLuc activities were measured 24 h post-transfection, and data are presented as RLU. Values shown are the mean (−SEM) of three independent experiments, each performed in duplicate. Statistical analysis was performed by an ordinary one-way ANOVA test (* *P* < 0.05).

Stau1^55^ overexpression extends the G2 phase of the cell cycle (46,55,74). Also, Stau1^55^ binds to the TAR stem-loop (SL) of the HIV-1 vRNA, enhancing cap-dependent translation initiation (33). As the HIV-1 IRES function is G2/M dependent (16,27), Stau1^55^ could enhance HIV-1 IRES activity by binding the TAR or by extending the G2 phase of the cell cycle. Therefore, we determined the impact of Stau1^55^ on the HIV-1 IRES that lacks TAR and the poly(A) SL (Figure 4A). The reporter plasmid dl HIV-1 IRES 104–336 shares all features with the dl HIV-1 IRES plasmid (Figures 2A and 4A), albeit it harbors only nts 104–336 of the pNL-4.3 5′UTR in its intercistronic region (16). HEK 293T cells were therefore transfected with the dl HIV-1 IRES or with the dl HIV-1 IRES 104–336 plasmids, and as expected, based on earlier reports (16,18), the RLuc and FLuc activities from the dl HIV-1 IRES 104–336 and dl HIV-1 IRES mRNA were comparable in magnitude (Figure 4B, left and middle panel). These results indicated that the 5′deletion mutant IRES (104–336) was functional in HEK 293T cells. However, in concordance with what was reported in HeLa and Jurkat T cells (16,18), translational activity driven by the full-length 5′UTR (1−336) was significantly higher than that obtained with the 5′deletion mutant IRES (104–336) in HEK 293T (Figure 4B, right panel).

**Figure 5.**
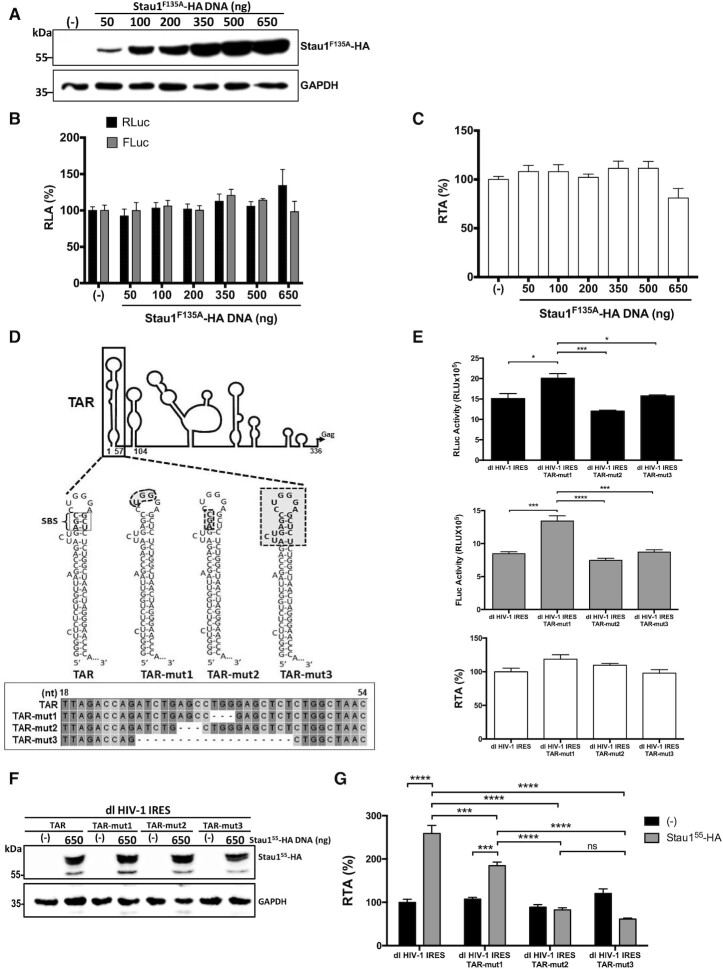
Staufen1-RNA interaction is required to promote HIV-1 IRES activity. HEK 293T cells were cotransfected with the dl HIV-1 IRES (300 ng) and different quantities (50–650 ng) of a plasmid encoding for a Stau1^55−^F135A-HA protein. (**A**) The overexpression of Stau1^55−^F135A-HA protein was confirmed by western blot using an anti-HA antibody and GAPDH as a loading control. (**B, C**) RLuc and FLuc activities were measured 24 h post-transfection, and results are presented as RLA (**B**) or RTA (**C**). The RLA and RTA values obtained in the absence (−) of the Stau1^55−^F135A-HA expressing plasmid were set to 100%. Values shown are the mean (±SEM) of four independent experiments, each performed in duplicate. Statistical analysis was performed by an ordinary one-way ANOVA test. (**D**) Schematic representation of the TAR-deletion mutants generated for the study based on mutants used to study Stau1^55^-TAR interaction (33). TAR corresponds to the wild-type RNA structure. The Staufen1 binding site (SBS) is highlighted (33). The highlighted areas in TAR-mu1, TAR-mut2, and TAR-mut3 correspond to the deleted nts. Mutants were verified by sequencing. (**E**) HEK 293T cells were transfected with the dl HIV-1 IRES, dl HIV-1 IRES TAR-mut1, dl HIV-1 IRES TAR-mut2, or dl HIV-1 IRES TAR-mut3 (300 ng) plasmids. RLuc (upper panel) and FLuc (middle panel) activities were measured 24 h post-transfection, and data are presented as RLU or as RTA (lower panel). (**F, G**) HEK 293T cells were cotransfected with the dl HIV-1 IRES, dl HIV-1 IRES TAR-mut1, dl HIV-1 IRES TAR-mut2, or dl HIV-1 IRES TAR-mut3 (300 ng) and 650 ng of a plasmid encoding for a Stau1^55^-HA_3_ protein. (**F**) The overexpression of Stau1^55^-HA_3_ was confirmed by western blot using an anti-HA antibody and GAPDH as a loading control. (**G**) RLuc and FLuc activities were measured 24 h post-transfection, and results are presented as RTA were the RTA obtained with the dl HIV-1 IRES in the absence of Stau1^55^-HA_3_ was set to 100% (±SEM). Values shown are the mean (±SEM) of three independent experiments, each performed in duplicate. Statistical analysis was performed by an ordinary one-way ANOVA test.

Next, the dl HIV-1 IRES 104–336 was cotransfected in HEK 293T cells together with increasing concentrations (50–650 ng) of the Stau1^55^-HA_3_ encoding plasmid. The overexpression of Stau1^55^-HA_3_ protein was confirmed by western blot using an anti-HA or anti-Staufen1 antibody and GAPDH as a loading control (Figure 4C). The RLuc and FLuc activities obtained when the dl HIV-1 IRES 104–336 was cotransfected in HEK 293T with the control DNA, not expressing Stau1^55^, were set to 100% (Figure 4D). Luciferase activities, expressed as RLA (Figure 4D), showed that the overexpression of Stau1^55^-HA_3_ (50–650 ng DNA) equivalently increased RLuc and FLuc by about 50% (Figure 5D), as before (Figure 2J), most probably due to RNA stabilization (34). However, the FLuc/RLuc ratio (i.e., RTA) showed that Stau1^55^-HA_3_ did not impact IRES activity from the dl HIV-1 IRES 104–336 reporter (Figure 4E). As an additional control, HEK 293T cells were transfected with the dl HIV-1 IRES 1–104 (300 ng) with (+), or without (−), the Stau1^55^-HA_3_ expressing plasmid (650 ng). The reporter plasmid dl HIV-1 IRES 1–104 shares all features with the dl HIV-1 IRES and dl HIV-1 104–336 plasmids (16), except that its intercistronic region comprises nts 1–104 of the pNL-4.3 vRNA 5′UTR, including only the TAR and Poly(A) SL (Figures 2A and 4A), and lacks IRES activity in HeLa cells (16). As expected, only RLuc activity ($\sim$1.3 × 10^6^ RLU) was readily detected in HEK 293T cells transfected with the dl HIV-1 IRES 1–104, while FLuc activity ($\sim$5 × 10^3^ RLU) was considered at background levels (Figure 4F). Thus, in agreement with earlier results (16), the dl HIV-1 IRES 1–104 mRNA lacks IRES activity in HEK 293T cells. The co-transfection of dl HIV-1 IRES 1–104 with the Stau1^55^-HA expressing plasmid (650 ng) significantly increased RLuc activity ($\sim$52% increase), while FLuc activity ($\sim$5 × 10^3^ RLU) remained at background levels (Figure 4F). These observations (Figure 4), together with results presented in Figure 2, indicated that the upstream RNA elements (nt 1–104) of the 5′UTR of the HIV-1 vRNA, which alone lack IRES activity (Figure 4F and (16)), are required for Stau1^55^ to promote HIV-1 IRES activity in the context of the full-length 5′UTR (Figure 4D and E). Furthermore, as the Stau1^55^ binding site (SBS) is located in the TAR (33), our results also suggest that Stau1^55^-vRNA interaction is necessary to stimulate IRES activity, indicating that Stau1^55^ acts as an ITAF for the HIV-1 IRES.

### Staufen1−RNA interaction is required to promote HIV-1 IRES activity in HEK 293T cells

Stau1^55^ contains four dsRNA-binding domains (dsRBD) consensus sequences, of which the dsRBD3 possesses a strong dsRNA-binding activity, while the dsRBD4 exhibits somewhat weaker binding capacity (38,47). Stau1^55^ has two additional RBDs, RBD2 and RBD5, but neither bind RNA (38,47). The dsRBD3 was shown to primarily mediate the Stau1^55^-HIV-1 TAR RNA and Gag interactions (32,33). To further confirm whether vRNA binding by Stau1^55^ is required to stimulate HIV-1 IRES activity, the dl HIV-1 IRES plasmid was cotransfected in HEK 293T cells together with different concentrations (50–650 ng) of a plasmid encoding for an HA_3_-tagged Stau1^55^-F135A mutant protein or a control DNA. Stau1^55^-F135A-HA_3_ possesses a Phe-to-Ala mutation at position 135 in the dsRBD3 domain that leads to a loss in its dsRNA-binding ability (32,33,35,47). The expression of Stau1^55^-F135A-HA_3_ protein was confirmed by western blot (Figure 5A). The RLuc and FLuc activities were measured, and data were expressed as RLA (Figure 5B) or RTA (Figure 5C). The overexpression of Stau1^55^-F135A-HA_3_ did not affect RLuc or FLuc activities of the dl HIV-1 IRES mRNA. Hence, the dsRBD3 mutant protein Stau1^55^-F135A-HA_3_ was unable to promote HIV-1 IRES activity in HEK 293T cells (Figure 5C). These observations suggest that the dsRBD3 of Stau1^55^ is responsible for mediating the enhancement of HIV-1 IRES activity in HEK 293T cells. Additionally, these observations further indicate that Stau1^55^ binding to the RNA is required for its function of stimulating the HIV-1 IRES.

Stau1^55^ binds the TAR-RNA at the SBS with high affinity (*K*_d_ 3.5 nM) (33). The SBS is located in the upper stem between the bulge and the loop as determined by filter binding and Northwestern using purified Stau1^55^ and ^32^P-labeled TAR-RNA (33) (Figure 5D). Mutation in the SBS sharply impacts Stau1^55^-vRNA interaction (*K*_d_ 872.5 nM) (33). To further validate our previous observations (Figures 4 and 5A-C), three dl HIV-1 IRES bicistronic vectors harboring different TAR-deletion mutations (TAR-mut) were generated. The selection of TAR-mut was based on TAR-substitution mutants characterized in previous studies of Stau1^55^-TAR interaction (Figure 5D) (33). HEK 293T cells were transfected with the dl HIV-1 IRES and the TAR-mut's harboring dl HIV-1 IRES plasmids. The RLuc and FLuc activities from all dl HIV-1 IRES plasmids were comparable in magnitude (Figure 5E, upper and middle panel). Nonetheless, RLuc and FLuc activities from the dl HIV-1 IRES TAR-mut1 plasmid were significantly higher than the rest (Figure 5E). However, the FLuc/RLuc ratio (RTA) analysis revealed no significant differences among the vectors, suggesting that IRES activity in HEK 293T cells was equivalent for all (Figure 5E, lower panel). Next, the dl HIV-1 IRES and the TAR-mut plasmids were cotransfected in HEK 293T cells together with the Stau1^55^-HA_3_ expressing plasmid (650 ng) or a control DNA. The overexpression of Stau1^55^-HA_3_ protein was confirmed by western blot (Figure 5F). Luciferase activities were measured, and the RTA of the dl HIV-1 IRES plasmid cotransfected with the control DNA (−), not expressing Stau1^55^, was set to 100% (Figure 5G). In the absence of Stau1^55^-HA_3_, the IRES activity of the dl HIV-1 IRES and dl HIV-1 IRES TAR-mut plasmids was equivalent (Figure 5G, black bars). As expected, we found that expression of Stau1^55^-HA_3_ significantly increased ($\sim$160%) HIV-1 IRES activity from the dl HIV-1 IRES reporter. IRES activity from dl HIV-1 IRES TAR-mut1, harboring a deletion in the apical RNA loop, was also significantly increased ($\sim$77%), yet to lower levels than the no-mutated HIV-1 IRES, by the expression of Stau1^55^-HA_3_ (Figure 5G). This observation is consistent with northwestern assays showing that mutations in the apical loop reduce, but do not abolish, Stau1^55^ binding to the TAR RNA (33). Confirming our previous results (Figures 4 and 5A−C), overexpression of Stau1^55^ did not impact IRES activity of the dl HIV-1 IRES TAR-mut2, lacking the SBS, or the dl HIV-1 IRES TAR-mut3 lacking the bulge, the upper stem, and the loop (Figure 5D and G). Together these findings confirm that Stau1^55^-vRNA interaction is required for Stau1^55^ to stimulate the activity of the HIV-1 IRES.

### Staufen1 and the Staufen-dsRBD3-domain rescues HIV-1 IRES activity in Staufen1 knockout cells

**Figure 6.**
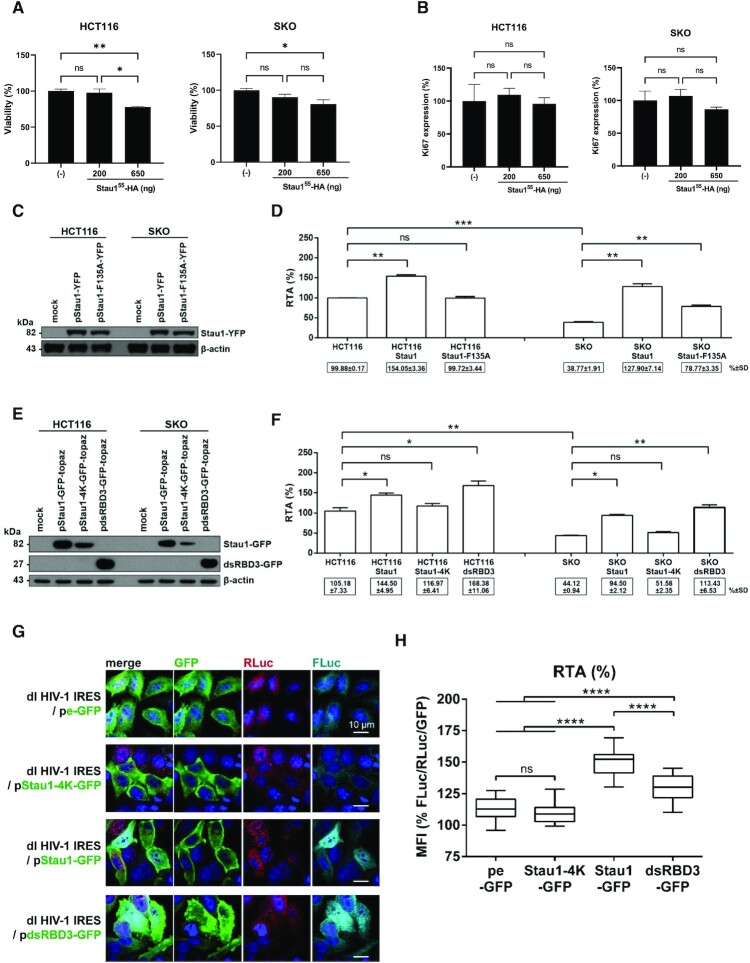
Staufen1 and Staufen1-dsRBD3 rescues HIV-1 IRES activity in Staufen1 knockout HCT116 and HeLa cells. HCT116 or Staufen1 knockout HCT116 (SKO) cells were transfected, or not, with the Stau1^55^-HA_3_ expression plasmid (200 or 650 ng), and cell viability (**A**) and cell proliferation (**B**) were analyzed by flow cytometry as indicated in Material and Methods. (**C, D**) The dl HIV-1 IRES plasmid (300 ng) was cotransfected, or not, with the Stau1^55^-YFP expression construct was transfected into HCT116 or SKO cells. (**C**) The overexpression of the Stau1^55^-YFP and Stau1^55^-F135A-YFP in transfected HCT116 and SKO cells was confirmed by western blot using an anti-GFP antibody and β-actin as a loading control. (**D**) RLuc and FLuc activities were measured 24 h post-transfection, and results are presented as RTA. The RTA obtained for HCT116 cells transfected with dl HIV-1 IRES in the absence of Stau1^55^-HA_3_, or the mutant protein was set to 100%. Statistical analysis was performed using an unpaired two-tailed *t*-test (ns, non-significant; * *P* ≤ 0.05; ** *P* ≤ 0.01; *** *P* ≤ 0.001; **** *P* ≤ 0.0001). (**E, F**) The dl HIV-1 IRES plasmid was cotransfected, or not, with the Stau1^55^-GFP-topaz, mutant Stau1^55^-4K-GFP-topaz or Stau1^55^-dsRBD3-GFP-topaz expression construct into HCT116 or SKO cells. (**E**) The expression of Stau1^55^-GFP-topaz, Stau1^55^-4K-GFP-topaz, and Stau1^55^-dsRBD3-GFP-topaz was confirmed by western blot using an anti-GFP antibody and β-actin as a loading control. (**F**) RLuc and FLuc activities were measured 24 h post-transfection, and results are presented as RTA. The RTA obtained for the HCT116 cells that did not overexpress Stau1^55^-recombinant proteins or any of its domains was set to 100%. Statistical analysis was performed using an unpaired two-tailed *t*-test (ns, non-significant; * *P* ≤ 0.05; ** *P* ≤ 0.01; *** *P* ≤ 0.001; **** *P* ≤ 0.0001). (**G, H**) HeLa cells were cotransfected with the dl HIV-1 IRES plasmid together with the pe-GFP control plasmid, Stau1^55^-4K-GFP-topaz, Stau1^55^-GFP-topaz, or Stau1^55^-dsRBD3-GFP-topaz expression constructs. (**G**) Immunofluorescence imaging demonstrating expression and localization of peGFP, Stau1^55^-4K-GFP-topaz, Stau1^55^-GFP-topaz, and Stau1^55^-dsRBD3-GFP-topaz (green) in addition to that of RLuc (red) and Fluc (cyan) in HeLa cells cotransfected with the dl HIV IRES plasmid. (**H**) Graphical representation of results shown in panel (**G**), calculating [RTA/GFP] from imaging data using the mean fluorescence intensity (MFI) values obtained for FLuc, RLuc, and GFP. Statistical analysis was performed using a one-way ANOVA with Tukey post-test for multiple comparisons. ** *P* ≤ 0.01; **** *P* ≤ 0.0001.

The above results suggest that Stau1^55^ alone binds to the TAR RNA and promotes HIV-1 IRES activity. Next, we sought to validate these observations in CRISPR-Cas9-edited Staufen1 knockout HCT116 (SKO) cells, depleted of both Stau1^55^ and Stau1^63^ (36,60). Furthermore, HCT116 cells support HIV-1 replication upon pNL-4.3 transfection (36). As a first step HCT116 and SKO cells were transfected with the plasmid expressing Stau1^55^-HA_3_ (200 and 650 ng). Overexpression of Stau1^55^-HA_3_ at the highest concentration tested (650 ng of plasmid) reduced HCT116 (by $\sim$22%) and SKO (by $\sim$16%) cell viability, but not at lower DNA quantities (Figure 6A). Stau1^55^-HA_3_ overexpression did not affect HCT116 or SKO cell proliferation at any concentration tested (Figure 6B). Next, HCT116 or SKO cells were transfected with the dl HIV-1 IRES plasmid alone (control DNA) or in combination with plasmids (500 ng) encoding a yellow fluorescent protein (YFP)-tagged Stau1^55^ (Stau1^55^-YFP) or Stau1^55^-F135A-YFP protein. The expression of Stau1^55^-YFP and Stau1^55^-F135A-YFP proteins was confirmed by western blot using an anti–GFP antibody and β-actin as a loading control (Figure 6C). Luciferase activities were measured, and results presented as RTA showed that the expression of Stau1^55^-YFP in HCT116 cells significantly enhanced ($\sim$54% increase) HIV-1 IRES activity (Figure 6D). In contrast, the expression of the dsRNA-binding mutant Stau1^55^-F135A-YFP had no impact on the activity of the HIV-1 IRES (Figure 6D). These results recapitulate those obtained in HEK 293T cells using HA-epitope tagged Stau1^55^ (Figures 2 and 5). HIV-1 IRES was significantly attenuated ($\sim$61%) but not abolished in Staufen1 knockout SKO cells compared to parental HCT116 cells (Figure 6D), confirming that Stau1^55^ plays a role in promoting IRES activity (Figures 4 and 5). In rescue experiments in SKO cells, the overexpression of Stau1^55^-YFP restored and even enhanced (∼90% increase) HIV-1 IRES activity compared to non-transfected SKO cells (Figure 6D). The expression of Stau1^55^-F135A-YFP in SKO cells also increased HIV-1 IRES activity ($\sim$40% increase) compared to non-transfected SKO cells (Figure 6D). This observation suggested that in contrast to HEK 293T cells (Figure 5A-C), in SKO cells, the dsRBD4 might also contribute to HIV-1 IRES activity (Figure 6D, see below). Nonetheless, HIV-1 IRES activity remained reduced ($\sim$21% lower) in SKO cells expressing Stau1^55^-F135A-YFP compared to HCT116 cells (Figure 6D). These results show that Stau1^55^-YFP, but not the mutant Stau1^55^-F135A-YFP, fully rescues HIV-1 IRES activity in SKO cells, confirming the role of Stau1^55^ as an ITAF of the HIV-1 IRES.

The molecular behavior of dsRBD3, including its ability to interact with its targets RNAs and ribosomes, is maintained even when isolated from the rest of the protein (47,58,81,82). We queried whether the expression of Stau1^55^-dsRBD3 alone could impact HIV-1 IRES activity in SKO cells. For this, HCT116 or SKO cells were transfected with the dl HIV-1 IRES plasmid alone or together with plasmids expressing Stau1^55^-GFP-topaz, Stau1^55^-dsRBD3-GFP-topaz, or mutant Stau1^55^-4K-GFP-topaz harboring point mutations in the dsRBD3 and dsRBD4, abrogating both RNA binding domains (59). The overexpression of the Stau1^55^ and the Stau1^55^-dsRBD3 domain was confirmed by western blot using an anti–GFP antibody and β-actin as a loading control (Figure 6E). Luciferase activities were measured, and results are presented as RTA (Figure 6F). As previously observed (Figure 6D), in this new series of experiments, HIV-1 IRES activity was considerably reduced ($\sim$61%) in SKO cells when compared to HCT116 cells (Figure 6F). The expression of Stau1^55^-GFP-topaz significantly enhanced HIV-1 IRES activity in HC116 cells ($\sim$40% increase), while the mutant Stau1^55^-4K-GFP-topaz did not affect HIV-1 IRES activity (Figure 6F). In SKO cells, Stau1^55^-GFP-topaz, but not Stau1^55^-4K-GFP-topaz, restored HIV-1 IRES activity to levels comparable to that found in HCT116 cells (Figure 6F). This observation confirmed that Stau1^55^ binding to the vRNA is a requirement to promote HIV-1 IRES-mediated translation. Results obtained with Stau1^55^-F135A (Figure 6D) and Stau1^55^-4K-GFP-topaz (Figure 6F) confirm that the dsRBD4 also contributes to the ability of Stau1^55^ to enhance HIV-1 IRES activity in SKO cells. Strikingly, when expressed alone, the Stau1^55^-dsRBD3 domain alone significantly enhanced HIV-1 IRES activity ($\sim$63% increase) in HCT116 cells while again restoring HIV-1 IRES activity in SKO cells (Figure 6F). Together, these results indicate that Stau1^55^, but not Stau1^55^-F135A or Stau1^55^-4K, fully rescues HIV-1 IRES activity in SKO cells, confirming the role of Stau1^55^ as an ITAF of the HIV-1 IRES. These results also suggest that the Stau1^55^-dsRBD3 domain alone is sufficient to restore HIV-1 IRES activity in SKO cells.

We next sought to validate the impact of the dRBD3 domain of Stau1^55^ on HIV-1 IRES activity in a different cell line and by using an immunofluorescence (IF)-based approach to evaluate protein expression. As HEK 293T cells have limited cytoplasmic space (37,38,59), they were not considered for the assay. HeLa cells were selected as their cytoplasm is suitable for IF analysis, and they support pNL-4.3 replication (Figure 1) and HIV-1 IRES activity (16,24,27,28). HeLa cells were transfected with the dl HIV-1 IRES DNA together with the pStau1^55^-GFP-topaz, mutant pStau1^55^-4K-GFP-topaz or pStau1^55^-dsRBD3-GFP-topaz expressing plasmids. In these assays, the pStau1^55^-4K-GFP-topaz, which does not bind RNA or promote HIV-1 IRES activity ((59) and Figure 6G), and the empty plasmid expressing only eGFP (pe-GFP), were used as a negative control. The expression of the wild-type and mutant Stau1^55^ proteins and the dsRBD3 domain alone was confirmed by detecting GFP expression by IF in cells (Figure 6G). RLuc and FLuc reporter proteins were also detected in HeLa cells by IF as indicated in Materials and Methods. As anticipated, both the cap-dependent RLuc and the HIV-1 IRES-dependent FLuc reporter proteins could be readily detected by IF in HeLa cells transfected with the dl HIV-1 IRES plasmid (Figure 6G). The co-expression of RLuc, FLuc, and pe-GFP, pStau1^55^-GFP-topaz, mutant pStau1^55^-4K-GFP-topaz or pStau1^55^-dsRBD3-GFP-topaz in cells was also confirmed by IF (Figure 6G). The mean fluorescence intensity (MFI) values for RLuc and FLuc obtained from the imaging data were used to calculate the RTA, which were normalized to GFP expression to account for Stau1^55^ and dsRBD3 recombinant protein levels. The Stau1^55^-4K mutant normalized RTA value was set to 100%. Consistent with our observations in HEK 293T, HCT116, and SKO, the overexpression of pStau1^55^-GFP-topaz enhanced HIV-1 IRES activity in HeLa cells (Figure 6H). Also, confirming our findings in HCT116 and SKO cells (Figure 6E and F), the expression of Stau1^55^-dsRBD3-GFP-topaz significantly stimulated HIV-1 IRES activity in HeLa cells (Figure 6H). These observations confirm that the Stau1^55^-dsRBD3 alone is sufficient to promote HIV-1 IRES activity in cells, but the maximum stimulation in HeLa cells is obtained with the whole protein.

## DISCUSSION

The initiation of HIV-1 vRNA translation is achieved by alternative mechanisms (8,9,13–16,18,27). Both cap-dependent and IRES-mediated translation initiation of the HIV-1 vRNA are tightly regulated by host proteins (8,9). For example, cap-dependent translation initiation of the HIV-1 vRNA leading to Gag synthesis is reduced by hnRNPE1 (83), while it is enhanced by SRp40, SRp55 (84), UPF1 (75), TRBP (85,86), La autoantigen (87,88), the RNA helicases DEAD (Asp-Glu-Ala-Asp) box polypeptide 3 (DDX3) and DHX9 (14,89,90) and Stau1^55^ (33). Likewise, IRES-mediated translation of the HIV-1 vRNA relies on ITAFs (8,18,27), most of which are cellular RNA-binding proteins capable of forming ribonucleoproteins (RNP) (4,8,9). Interestingly, apart from their role in IRES-mediated translation initiation, most known HIV-1 ITAFs are also involved in other aspects of RNA metabolism, including RNA splicing, export, and stability (4,8,9). It has been proposed that ITAFs, together with the 5′UTR of the HIV-1 vRNA, assemble into a functional translational-RNP complex, having the ability to drive internal initiation (8,18,27). In this context, identifying proteins that bind the vRNA and regulate non-canonical translation initiation will further our understanding of the regulation of HIV-1 gene expression. Several features suggested that Stau1^55^ could act as a *bona fide* ITAF for the HIV-1 IRES. For example, Stau1^55^ binds to the TAR region of the HIV-1 vRNA, stimulating translation (33), and its depletion in HIV-1-expressing cells reduces the expression of the Gag protein without any impact of vRNA abundance (31,53).

In cells, Staufen1 resides in several dynamic RNP complexes (34,40,41). In HIV-1-expressing cells, Staufen1 binds the vRNA and Gag forming different Staufen1-containing complexes (31–36,52,72). We initiated this study by confirming that Staufen1 associates with the vRNA and Gag in HIV-1-expressing cells and validating that the protein's depletion reduced Gag protein synthesis (Figure 1). The reduction in Gag expression when Staufen1 was depleted using a siRNA strategy was not unexpected (31,33,53). Even so, by using a dl-RNA strategy, we show, for the first time, that the Stau1^55^ isoform binds the vRNA and modulates HIV-1 IRES activity (Figures 2, 4–6). Strikingly, Stau1^55^ emerges as a unique multifunctional translational regulator of HIV-1 vRNA gene expression, controlling both cap- and IRES-dependent translation initiation ((31,33,53) and Figures 2 and 6). How Stau1^55^ accomplishes the task of regulating two completely different mechanisms of translation initiation remains unknown. However, binding of Stau1^55^ to the HIV-1 vRNA was required to establish control over both cap- and IRES-mediated translation initiation ((33) and Figures 4–6). For HIV-1 IRES activity, we show that Stau1^55^ does not stimulate the activity of a 5′deletion mutant HIV-1 IRES (nts 104–336) (Figure 4), lacking the TAR and poly(A) stem–loop. Equally, Stau1^55^ does not stimulate the activity of an HIV-1 IRES lacking the SBS ((33) and Figure 5). Thus, to stimulate HIV-1 IRES activity, the Stau1^55^-TAR (SBS) interaction must be attained. Consistent with this conclusion, the Stau1-F135A or Stau1-4K mutants that are restricted in their ability to bind RNA did not promote HIV-1 IRES activity (Figures 5 and 6). As previously indicated (45), the number of Stau1^55^ molecules within a cell is fewer than that of ribosomes, suggesting that Stau1^55^ would favor translation of only a subpopulation of specialized mRNAs it interacts with, including the HIV-1 vRNAs, characterized for having a SBS within their UTRs. Consistent with this proposed target specificity, Stau1^55^ is known to interact with the HIV-1 vRNA uniquely but not with the spliced HIV-1 RNA species (32). The role of Stau1^55^ in translation is complex and highly influenced by the position at which the protein is recruited onto the mRNA. When bound to the 3′UTR of its target cellular mRNA Staufen1 induces Staufen1-mediated mRNA decay (44). However, our data shows that when attached to the 5′UTR of the HIV-1 vRNA (Figures 2, 4–6) or mRNAs containing the HIV-1 TAR-SL (33), Staufen1 stimulates translation.

It is tempting to speculate that the function of Stau1^55^ as a translational regulator is associated with the natural structural plasticity exhibited by the protein and its ability to induce local RNA structural modification due to its dsRNA binding activity (54,81,82). Also, the ability of Stau1^55^ to regulate cap- and IRES-mediated translation initiation of the HIV-1 vRNA could be linked either to the Stau1^55^-binding partners present within the translating Stau1^55^-HIV-1 RNP or to the post-translational modifications (PTMs) of individual protein partners of the RNP, including Stau1^55^ itself (25,31,34,69). This possibility is highly attractive as it would suggest that the Stau1^55^-HIV-1 RNP complex would be capable of sensing the environment adapting to favor either cap- or IRES-dependent translation initiation as previously proposed (12,13,20). This would allow viral gene expression to rapidly adapt to physiological changes induced either by the cellular antiviral response or by the viral replication cycle. Even though further studies are required to prove this possibility entirely, several reports already support the notion (12,13,20,22,91). For example, during HIV-1 infection, the viral protein R (Vpr) induces a cell cycle arrest in G2/M (13,92), a cell cycle stage known to compromise canonical cap-dependent translation initiation severely (18,91). Yet, in G2/M, transcription (93) and translation (13,16,27) of the HIV-1 vRNA are promoted. Staufen1 expression is also high in G2/M (55) and is enhanced when cap-dependent translation initiation is compromised (50). Furthermore, the expression of Staufen1 extends the G2 phase of the cell cycle (46,55,74). Therefore, it is expected that Stau1^55^ is a key player and a constitutive part of the RNP-complex modulating HIV-1 IRES activity during G2/M.

Another point to consider, but not directly evaluated in this study, is that Stau1^55^ binds to ribosomes (37,45,47). Specifically, Stau1^55^ interacts with the 60S ribosomal subunit (45). Thus, we cannot exclude the possibility that the function of Stau1^55^ or the Stau1^55^-HIV-1 RNP complex is to stabilize ribosome assembly, polysome association, and ribosome occupancy of the HIV-1 vRNA (33,47,64), all processes independent from the mechanism by which the HIV-1 vRNA recruits the 40S ribosomal subunit. This last possibility could explain why Stau1^55^ enhanced both cap- and IRES-mediated translation initiation as its function would be shared regardless of the mechanism of translation initiation.

As observed for Stau1^55^, overexpression of the Stau1^55^-dsRBD3 moiety alone was sufficient to promote HIV-1 IRES activity in cells expressing endogenous levels of Staufen1, HCT116, and HeLa, and rescue HIV-1 IRES activity in SKO cells (Figure 6). These findings support the possibility that, at least in part, RNA structural rearrangement induced by the dsRBD3 binding could assist in stimulating IRES-mediated translation. Studies of the *Drosophila* dStau1-dsRBD3, which is homologous to the human Stau1^55^-dsRBD3, showed that dsRBD3 binds hairpin RNA structures with double-helical stems with micromolar affinity (81). Binding of the dStau1–dsRBD3 to its target RNA induced a kink at the stem-loop junction, bending the RNA (81). It is plausible that this RNA structural change associated with dsRBD3 binding is responsible for enhancing HIV-1 IRES activity. Further experiments will be required to validate this last interpretation.

Despite the relevance of our findings, an apparent caveat of the study is that experiments were not conducted in T-cells, natural target cells for HIV-1. Nonetheless, previous reports conclude that the fundamental RNA-dependent mechanism-driving IRES function from the 5′UTR of the HIV-1 vRNA is similar between T-cells and other cell types, albeit with different activities (18). Furthermore, Stau1^55^ is ubiquitously expressed in different cell types where it plays a direct role in various stages of the HIV-1 replication cycle in all cells it has been evaluated, including T-cells, HeLa cells and HEK 293T, amongst others (32,34–37,52,72). Thus, we expect our results that show that Stau1^55^ enhances IRES-mediated translation initiation of the HIV-1 vRNA, to be valid in all cell lines and cell types capable of supporting HIV-1 IRES activity.

In conclusion, this study provides evidence that Stau1^55^ acts as a genuine ITAF for the HIV-1 IRES, thereby providing a novel and additional function of this multifunctional RBP, understood to regulate several steps of HIV-1 replication. The results described herein show that Stau1^55^ positively regulates HIV-1 vRNA cap-independent translation initiation.

## DATA AVAILABILITY

All data are available upon request.
