# Correction to ‘The double-stranded RNA-binding protein, Staufen1, is an IRES-transacting factor regulating HIV-1 cap-independent translation initiation’

**DOI:** 10.1093/nar/gkab1266

**Published:** 2021-12-15

**Authors:** Hade Ramos, Anne Monette, Meijuan Niu, Aldo Barrera, Brenda López-Ulloa, Yazmín Fuentes, Paola Guizar, Karla Pino, Luc DesGroseillers, Andrew J Mouland, Marcelo López-Lastra

**Affiliations:** Laboratorio de Virología Molecular, Instituto Milenio de Inmunología e Inmunoterapia, Departamento de Enfermedades Infecciosas e Inmunología Pediátrica, Escuela de Medicina, Pontificia Universidad Catóolica de Chile, Marcoleta 391, Santiago, Chile; HIV-1 RNA Trafficking Laboratory, Lady Davis Institute at the Jewish General Hospital, Montréal, Québec H3T 1E2, Canada; HIV-1 RNA Trafficking Laboratory, Lady Davis Institute at the Jewish General Hospital, Montréal, Québec H3T 1E2, Canada; Laboratorio de Virología Molecular, Instituto Milenio de Inmunología e Inmunoterapia, Departamento de Enfermedades Infecciosas e Inmunología Pediátrica, Escuela de Medicina, Pontificia Universidad Catóolica de Chile, Marcoleta 391, Santiago, Chile; Laboratorio de Virología Molecular, Instituto Milenio de Inmunología e Inmunoterapia, Departamento de Enfermedades Infecciosas e Inmunología Pediátrica, Escuela de Medicina, Pontificia Universidad Catóolica de Chile, Marcoleta 391, Santiago, Chile; Laboratorio de Virología Molecular, Instituto Milenio de Inmunología e Inmunoterapia, Departamento de Enfermedades Infecciosas e Inmunología Pediátrica, Escuela de Medicina, Pontificia Universidad Catóolica de Chile, Marcoleta 391, Santiago, Chile; HIV-1 RNA Trafficking Laboratory, Lady Davis Institute at the Jewish General Hospital, Montréal, Québec H3T 1E2, Canada; Department of Medicine, McGill University, Montréal, Québec H4A 3J1, Canada; Laboratorio de Virología Molecular, Instituto Milenio de Inmunología e Inmunoterapia, Departamento de Enfermedades Infecciosas e Inmunología Pediátrica, Escuela de Medicina, Pontificia Universidad Catóolica de Chile, Marcoleta 391, Santiago, Chile; Department of Biochemistry and Molecular Medicine, University of Montreal, P.O. Box 6128, Station Centre Ville, Montreal, Québec H3C 3J7, Canada; HIV-1 RNA Trafficking Laboratory, Lady Davis Institute at the Jewish General Hospital, Montréal, Québec H3T 1E2, Canada; Department of Medicine, McGill University, Montréal, Québec H4A 3J1, Canada; Laboratorio de Virología Molecular, Instituto Milenio de Inmunología e Inmunoterapia, Departamento de Enfermedades Infecciosas e Inmunología Pediátrica, Escuela de Medicina, Pontificia Universidad Catóolica de Chile, Marcoleta 391, Santiago, Chile

Errors were introduced in Figure 3 during production of the article (1). The X-axis of Figure 3D should be ΔSV40 not SV40. The publisher apologises for these errors and wishes to correct Figure 3 as shown below.

**Figure 3.**
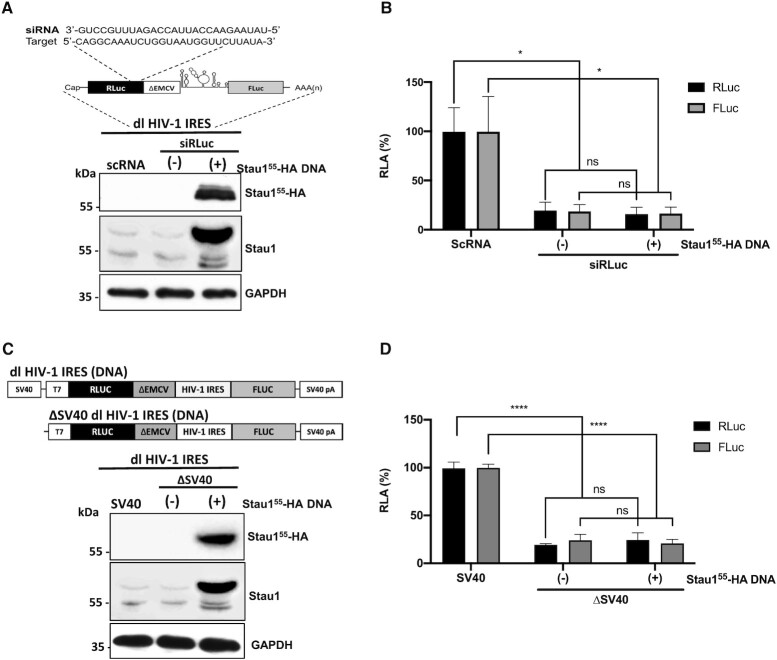
Staufen1 does not enhance alternative splicing of the dl HIV-1 IRES RNA nor increases the cryptic promoter activity of the dl HIV-1 DNA. (**A**, **B**) The dl HIV-1 IRES (150 ng) was cotransfected with a control scRNA (100 nM) or with siRLuc (100 nM), in the presence, or the absence (−), of the Stau155-HA3 (325 ng) plasmid. (**A**) Schematic representation of the dl reporter targeted by the siRNA RLuc (siRLuc) targeting the Renilla luciferase ORF (upper panel). Total protein extracts were prepared 48 hrs post-transfection. The expression of Stau155-HA3 was determined by western blot, using the GAPDHprotein as a loading control (lower panel). (**B**) RLuc and FLuc activities were measured and expressed relative to the values obtained with scRNA, set to 100% (RLA). Values shown are the mean (±SEM) for six independent experiments, each performed in duplicate. Statistical analysis was performed by an ordinary two-way ANOVA test (*P < 0.01; ns, not significant). (**C**, **D**) HEK 293T cells were transfected with either the dl HIV-1 IRES (150 ng) or a promoterless ΔSV40-dl HIV-1 IRES (150 ng) vector in the presence, or the absence (−), of the Stau155-HA3 (325 ng) plasmid. 24 hrs post-transfection total protein extracts were prepared. (**C**) Schematic representation of the dl HIV-1 IRES and ΔSV40-dl HIV-1 IRES plasmids (upper panel). The expression of Stau155-HA3 was determined by western blot, using the GAPDH protein as a loading control (lower panel). (**D**) RLuc and FLuc activities were measured, and results are expressed as RLA relative to the activities obtained from the dl HIV-1 IRES vector when in the absence of the Stau155-HA3, set to 100%. Values shown in are the mean (±SEM) for three independent experiments, each performed in duplicate. Statistical analysis was performed by an ordinary two-way ANOVA test (**** *P* < 0.0001; ns, not significant).

The published article has been updated. These corrections do not affect the results, discussion and conclusions presented in the article.
